# Detection of 3,4-Methylene Dioxy Amphetamine in Urine by Magnetically Improved Surface-Enhanced Raman Scattering Sensing Strategy

**DOI:** 10.3390/bios12090711

**Published:** 2022-09-02

**Authors:** Yue Wang, Xinyan Teng, Jiaying Cao, Yilei Fan, Xinling Liu, Xiaoyu Guo, Yu Xu, Ying Wen, Haifeng Yang

**Affiliations:** 1 The Education Ministry Key Lab of Resource Chemistry, Shanghai Key Laboratory of Rare Earth Functional Materials, Shanghai Municipal Education Committee Key Laboratory of Molecular Imaging Probes and Sensors and College of Chemistry and Materials Science, Shanghai Normal University, Shanghai 200234, China; 2 Key Laboratory of Drug Prevention and Control Technology of Zhejiang Province, Department of Criminal Science and Technology, Zhejiang Police College, Hangzhou 310053, China; 3 Key Laboratory of Drug Monitoring and Control of Zhejiang Province, National Anti-Drug Laboratory Zhejiang Regional Center, Hangzhou 310053, China

**Keywords:** 3,4-Methylene Dioxy Amphetamine, SERS, magnetically inducing sandwich structure, trace sensing

## Abstract

Abuse of illicit drugs has become a major issue of global concern. As a synthetic amphetamine analog, 3,4-Methylene Dioxy Amphetamine (MDA) causes serotonergic neurotoxicity, posing a serious risk to human health. In this work, a two-dimensional substrate of ITO/Au is fabricated by transferring Au nanoparticle film onto indium–tin oxide glass (ITO). By magnetic inducing assembly of Fe_3_O_4_@Au onto ITO/Au, a sandwich-based, surface-enhanced Raman scattering (SERS) detection strategy is designed. Through the use of an external magnet, the MDA is retained in the region of hot spots formed between Fe_3_O_4_@Au and ITO/Au; as a result, the SERS sensitivity for MDA is superior compared to other methods, lowering the limit of detection (LOD) to 0.0685 ng/mL and attaining a corresponding linear dynamic detection range of 5–10^5^ ng/mL. As an actual application, this magnetically improved SERS sensing strategy is successfully applied to distinguish MDA in urine at trace level, which is beneficial to clinical and forensic monitors.

## 1. Introduction

Abuse of illicit drugs poses significant adverse impact on human health and has also resulted in a serious social security problem [1,2] since the late 1970s. Recently, MDA as a synthetic amphetamine analog, which is structurally related to a wide variety of other naturally and synthetic compounds such as amphetamine (a psychomotor stimulant), catecholamines, and mescaline (a potent hallucinogen), has been an important issue of global concern [3]. MDA is included in category I of psychotropic drugs in China [4] and in schedule I of controlled substances in America [5], but is permitted to be used for medical purposes. As an actual situation, MDA is currently one of the most popular substances used recreationally in North America [6]. However, there are many clinical cases indicating that MDA would cause serotonergic neurotoxicity [7] due to stimulant effect on the central nervous system [8]. MDA is more toxic than its close congener, 3,4-methylenedioxy- methamphetamine (MDMA, “Ecstasy”) [9], and has significant addictive potential [10,11,12,13,14].

In forensic toxicology laboratories, high-performance liquid chromatography (HPLC) [15], gas chromatography/mass spectrometry (GC-MS) [16,17], liquid chromatography/mass spectrometry (LC-MS) [18], enzyme-linked immunosorbent assay (ELISA) [19], and electrochemistry [20,21] are currently used to detect drugs [22] in biological fluids such as urine [23,24,25], saliva [26], and plasma/blood [26]. However, these techniques are costly, time-consuming, and destructive [26], and consume reagents [27]. Simultaneously, as rapid and on-site portable methods, many biosensors have been widely explored. Very recently, Koklu et al. integrated alternating-current electrothermal flow in an organic electrochemical transistor to develop a portable label-free biosensor for ultrarapid, sensitive, and selective analysis of SARS-CoV-2 spike protein in human saliva [28]. Mirzajani et al. designed a printed circuit-board-based electrode and optimized an alternating current signal to quantitatively determine bisphenol for on-site, low-resource settings [29]. Therefore, developing a sensitive, selective, and on-site rapid biosensor for screening MDA is also a promising strategy in view of the drug’s continuing illegal distribution [30].

Surface-enhanced Raman scattering (SERS) spectroscopy with superior sensitivity and molecular structure fingerprint information has been extensively employed in the identification of controlled substances [31,32] such as opiates [33], amphetamines [34], benzodiazepines [35], and methamphetamine [36] at trace level by using confocal laser Raman systems [37]. With commercially available portable Raman spectrometers, SERS technology exhibits tremendous potential to become a quick and distinguishable assay for on-site, field testing of illicit drugs [38,39].

Fe_3_O_4_ coupled with Au or Ag nanoparticles (NPs) could realize rapid magnetic responsiveness [40] and optimize the SERS effect, which has commonly engaged considerable research interest in the field of analytical methods [41,42]. Magnetically induced accumulation is a greatly effective way to concentrate the amount of the target molecule, which benefits and promotes detection sensitivity [43]. We previously prepared a Au dotted magnetic network nanostructure (Au-MNN) [44] and presented a magnetically optimized SERS strategy to detect pesticide residues on vegetables at femtomole level, which was done by magnetically inducing the generation of a large surface plasmon resonance (SPR) field (termed “hot spots”) under laser exposure.

**Figure 1:**
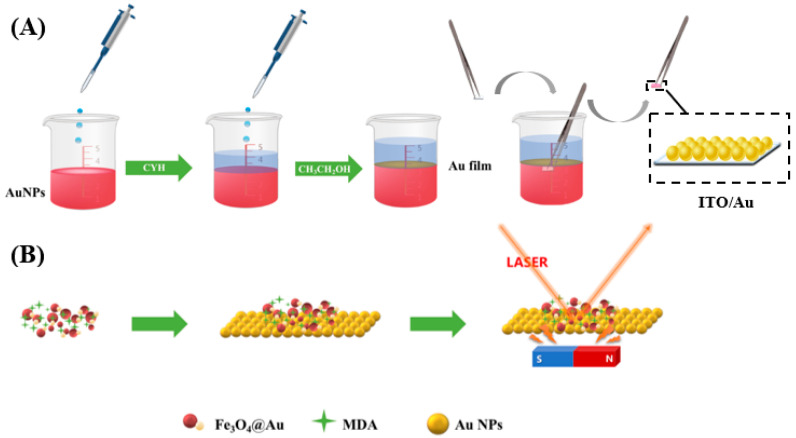
Schematic diagram of (**A**) self-assembly preparation of ITO/Au and (**B**) improvement of SERS detection sensitivity for MDA by magnetically inducing a sandwich structure.

In this work, by taking advantage of multiple benefits from the SERS technique and magnetic improvement, an SERS sensing platform to detect the MDA was designed. As shown in Figure 1, a two-dimensional substrate is constructed by transferring self-assembly film of Au NPs on the water–oil interface onto the ITO, designated as ITO/Au. Simultaneously, an Fe_3_O_4_@Au composite is prepared. For on-site detection, the sample solution containing MDA is mixed with Fe_3_O_4_@Au. Finally, with the assistance of an external magnet, the certain mixture of Fe_3_O_4_@Au and MDA is cast onto ITO/Au to form a sandwich structure for the SERS-sensitive detection. Because the MDA molecules are kept in the gap between Fe_3_O_4_@Au and ITO/Au, the SPR hotspots increase the SERS detection sensitivity to trace levels of MDA in human urine.

## 2. Experimental Methods

### 2.1. Materials

Inositol hexakisphosphate aqueous solution (IP_6_, 90%) was purchased from Sigma-Aldrich. FeCl_3_·6H_2_O (99%), FeCl_2_·4H_2_O (99%), NaOH (≥96.0%), sodium citrate (Na_3_C_6_H_5_O_7_·2H_2_O, 99.8%), and rhodamine 6G (R6G) were from Adamas Reagent. Cyclohexane (CYH, 99.5%) and absolute alcohol (CH_3_CH_2_OH) were obtained from Macklin Reagent (Shanghai, China). Chloroauric acid (HAuCl_4_·4H_2_O, 99.9%) was bought from Sinopharm Chemical Reagent (Shanghai, China). MDA was provided by Zhejiang Police College, Hangzhou. All reagents were used without further purification. All solutions were made with ultrapure water (18.25 MΩ cm), which was produced by using a Millipore water purification system.

### 2.2. Instrumentation

UV−visible absorption spectra were recorded with a UV−visible spectrophotometer (Shimadzu, Kyoto, Japan UV-1800). SERS spectra were collected by a Raman imaging microscope (Thermo DXR2xi, Madison, WI, USA). A field-emission scanning electron microscope (SEM, JEOL6380LV) and transmission electron microscopy (TEM, JEOL JEM-2000 FX) were used to observe the morphology of nanomaterials. The morphology and structures of ITO/Au substrate were characterized by an atom-force microscope (AFM, Bruker Dimension Icon). The magnetic properties of the observed nanocomposites were evaluated by using a vibrating sample magnetometer (VSM, Lake Shore VSM-736). Ultivo Triple Quadrupole LC/MS (Agilent, No.1 Yishun Ave 7, Singapore) and MassHunterC1.1 system were used to validate the SERS results.

### 2.3. Preparation of ITO/Au Two-Dimensional Substrate

Au NPs with an average size of 30 nm were prepared by citrate reduction according to Frens’ method [45]. In brief, 250 μL HAuCl_4_·4H_2_O (0.1 mol/L) was boiled for 10 min in 100 mL ultrapure water. Then, 1 mL of freshly prepared 1% sodium citrate solution was dripped rapidly and stirred for 30 min. The volume of Au NPs was concentrated to one-tenth of the original volume for later use. Briefly, as-prepared Au NPs solution was centrifuged at 6000 r/min for 10 min to obtain different Au NPs sols with 0-, 2-, 5-, 10-, and 20-fold concentrations by adding required volumes of ultrapure water. Then, 1 mL raw or concentrated Au NPs solution was injected into a 10 mL beaker, followed by adding 1 mL of CYH as the driving agent. After adding 1 mL of ethanol into the solution, a dense gold nanofilm with a metallic luster was formed by assembly at the interface of water/CYH. The gold film could be easily transferred from the water/CYH interface onto abluent ITO glass (5 mm × 10 mm), which had been pretreated in boiling activation solution (NH_3_·H_2_O:H_2_O_2_:H_2_O = 1:1:5 in *v*/*v*) for 30 min. Then, water and CYH were evaporated at room temperature.

### 2.4. Synthesis of Fe_3_O_4_@Au Nanoparticles

Fe_3_O_4_@Au nanoparticles were synthesized in accordance with our previously reported method [44]. In short, the mixture of 0.318 g FeCl_3_·6H_2_O and 0.130 g FeCl_2_·4H_2_O was dissolved in boiled IP_6_ ultrapure solution. After stirring for 1 h, 1.2 mL NaOH (0.4 mol/L) and another 5 mL IP_6_ was added successively into this mixture. The Fe_3_O_4_@Au nanoparticles were collected with a magnet, and again carefully rinsed and dispersed in the required volume of water. Then, 2.5 mL HAuCl_4_·4H_2_O (1% wt) was injected into the solution and 5 mL sodium citrate (1% wt) was rapidly added to the solution after refluxing for 15 min. After heating for 45 min, Fe_3_O_4_@Au nanoparticles were collected by using a magnet.

### 2.5. Magnetically Improved SERS Detection

Fe_3_O_4_@Au nanoparticles and MDA solution were fully mixed in a volume ratio of 1:2; then, 30 μL of the mixture was dropped to the surface of the ITO/Au with the assistance of a magnet under the ITO/Au. Raman spectra were recorded by using a DXR2xi Raman microscope with a 50× objective and excitation laser at 785 nm with 6.0 mW power. An acquisition time of 0.1 s was applied to avoid the heat effect of the laser on the sample; 1000 accumulations were used so that more target molecules would approach the vicinity of hotspots, thus obtaining a good SERS signal–noise ratio. The whole SERS detection time for each sample required 100 s.

### 2.6. Pretreatment of Actual Samples

One milliliter of urine sample was spiked with 10 μL MDA standard solution to mimic an actual sample. Before the Raman test, 1 mL methanol was added to the urine sample and given a full shake. The mixture was centrifuged at 6000 r/min for 10 min after showing white precipitate of proteins. Nitrogen was then purged to remove excess methanol. The final volume of residue was maintained at 1 mL for later experiments.

## 3. Results and Discussion

### 3.1. Characterization of Materials

**Figure 2:**
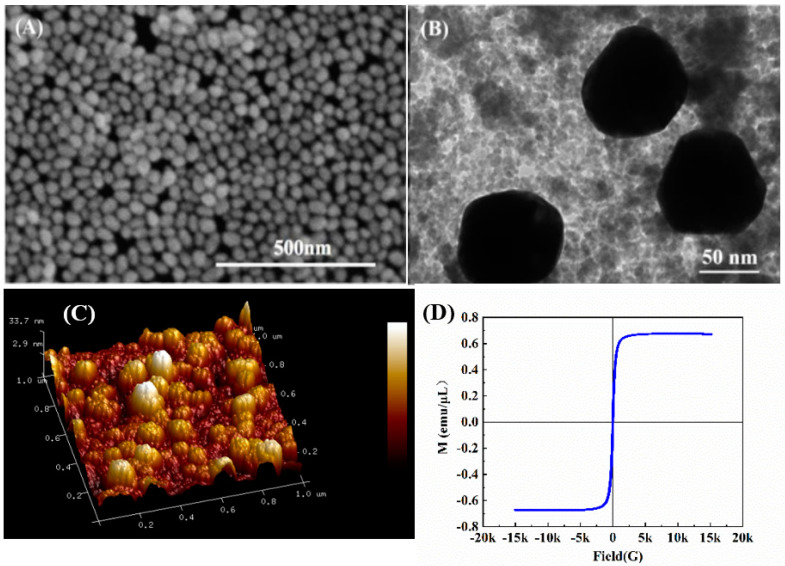
(**A**) SEM image of ITO/Au. (**B**) TEM image of Fe_3_O_4_@Au. (**C**) Three−dimensional AFM image of ITO/Au. (**D**) Hysteresis loop of Fe_3_O_4_@Au.

**Figure 3:**
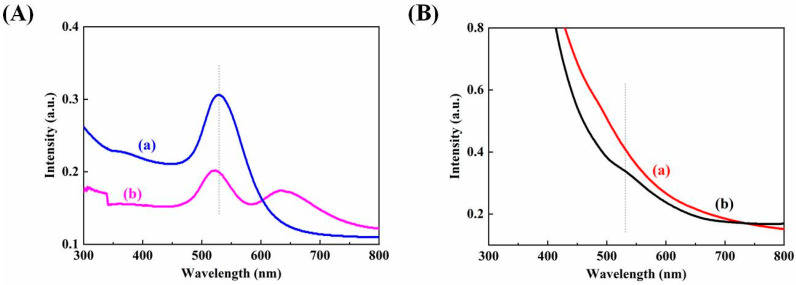
UV–visible spectra: (**A**) Au NPs (a) and ITO/Au (b); (**B**) Fe_3_O_4_ (a) and Fe_3_O_4_@Au (b).

Figure 2A,C show the SEM and AFM images of ITO/Au, respectively. Clearly, continuous film is successfully self-assembled by ~30 nm Au NPs over the whole surface of ITO in uniform distribution. The UV–visible spectra of pure Au NPs and ITO/Au are shown in Figure 3A. The novel peak at 650 nm of ITO/Au with respect to Au NPs means the certain aggregation of Au NPs in the assembly film to generate numerous SPR hot spots on the surface, which is very beneficial to amplification of SERS signals in the subsequent experiments.

The morphology of the Fe_3_O_4_@Au was characterized by TEM. In Figure 2B, Au NPs with an average size of about 80 nm could be bound to the magnetic network nanostructure of Fe_3_O_4_ (gray composite) via phosphate groups in dispersive way due to the presence of IP_6_. The Fe_3_O_4_@Au NPs were collected by a magnet and washed several times with ultrapure water to remove excess organic compounds. Additionally, the Raman scattering section of IP6 is quite small and has little effect on the SERS detection of MDA. The magnetic property of Fe_3_O_4_@Au was investigated with a vibrating sample magnetometer. As shown in Figure 2D, the curve with minor hysteresis loops indicates Fe_3_O_4_@Au has superior magnetic behavior. In Figure 3B, a featureless band of Fe_3_O_4_ nanoparticles and SPR band of Fe_3_O_4_@Au at 529 nm is visible, also confirming the formation of Fe_3_O_4_@Au.

### 3.2. Optimization of Self-Assembly for ITO/Au

**Figure 4:**
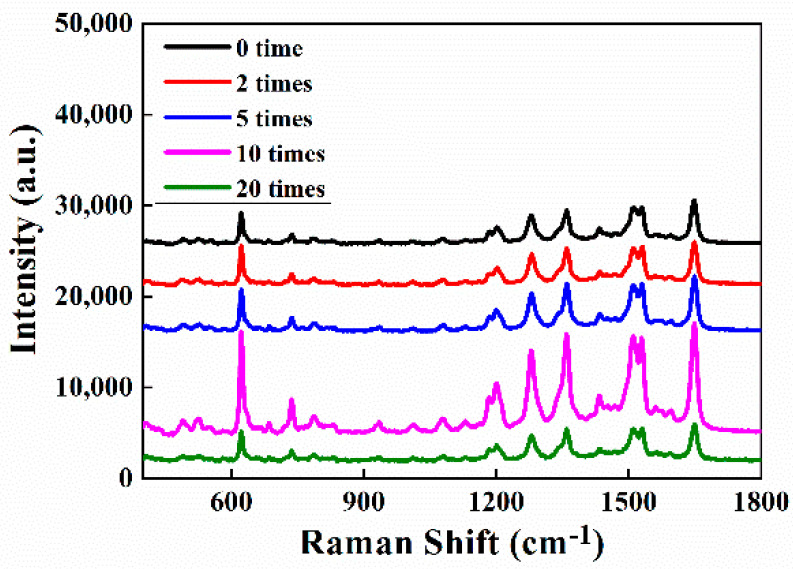
Raman spectra of R6G on ITO/Au prepared by using different concentrations of Au NP sols.

As noted above, the Au NPs monolayer in a large area was constructed by using a water/CYH interface. The reversible aggregation of Au NPs severely influences density of Au NPs in film, which is closely related to the SERS properties. In this work, different concentrations of Au NP sols were obtained by centrifugation and used for optimizing the assembly of Au nanofilms and finally preparing ITO/Au substrates. Figure 4 shows the SERS spectra of 10^−6^ mol/L R6G solution recorded for different ITO/Au substrates. It indicates that the strongest SERS signal can be achieved when Au sol is concentrated 10 times, for instance, 10 mL to 1 mL. It is due to formation of an imperfect monolayer of An NPs at the ITO surface, which sufficiently generates numerous hot spots.

### 3.3. SERS Performance of Sandwich Structure of ITO/Au and Fe_3_O_4_@Au

**Figure 5:**
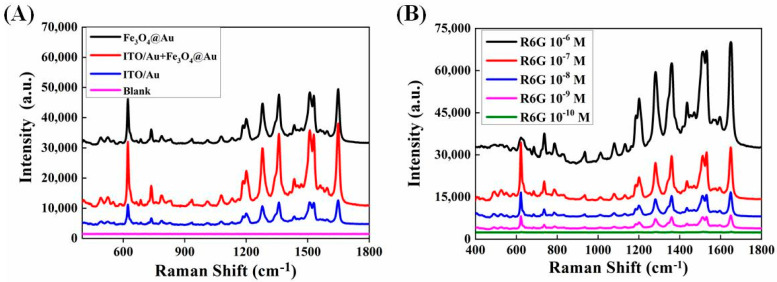
(**A**) Raman spectra of 10^−6^ mol/L R6G on optimal ITO/Au, Fe_3_O_4_@Au, and sandwich structure of ITO/Au and Fe_3_O_4_@Au. (**B**) Raman spectra of different concentrations of R6G on ITO/Au by using magnetically inducing strategy.

SERS spectra of 10^−6^ mol/L R6G on ITO/Au, Fe_3_O_4_@Au and sandwich structure of ITO/Au and Fe_3_O_4_@Au were acquired. As clearly shown in Figure 5A, the sandwich-structure-based detection strategy contributes the greatest enhancement. As indicated in Figure 5B, by magnetically inducing the sandwich structure, the limit of SERS detection of R6G can be as low as 10^−10^ mol/L.

**Figure 6:**
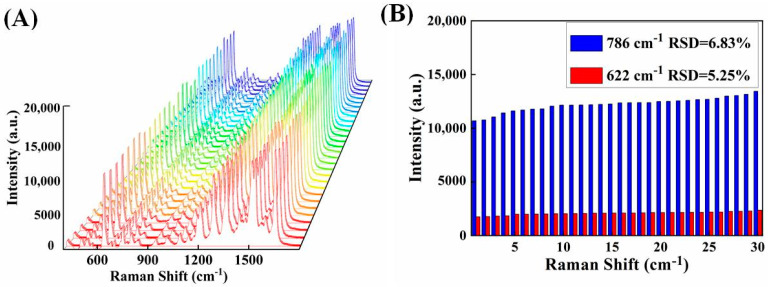
(**A**) Raman spectra of 30 randomly selected points on ITO/Au. (**B**) SERS peak intensity of 30 points was randomly selected on ITO/Au (786 and 622 cm^−1^).

The homogeneity and reproducibility of the Raman signals from the SERS substrate are crucial aspects in the subsequent detection. The SERS intensities of R6G (10^−6^ mol/L) at 30 random sites across the entire sandwich structure of ITO/Au and Fe_3_O_4_@Au are basically same as presented in Figure 6A. In Figure 6B, based on calculations using the typical Raman peaks at 622 and 786 cm^−1^, the relative standard deviations (RSDs) were of 5.25% and 6.83%, respectively, displaying good signal uniformity on 2D substrate. This benefits the subsequent qualitative detection of drugs.

**Figure 7:**
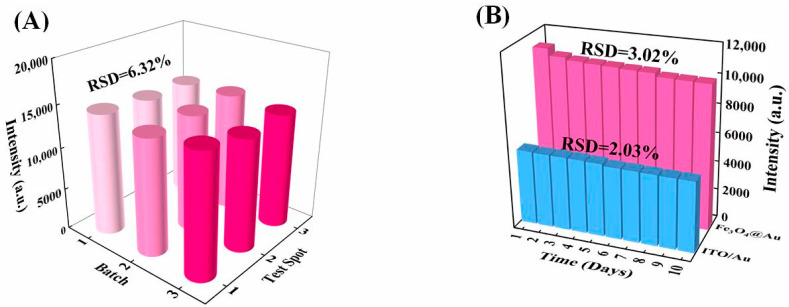
(**A**) SERS signals of R6G recorded from nine preparation batches of ITO/Au. (**B**) Storage stability check of R6G on ITO/Au and using Fe_3_O_4_@Au after 10 days.

For checking preparation reproducibility, the SERS intensities at 622 cm^−1^ of 10^−6^ mol/L R6G solution were recorded from three random points on one ITO/Au of three different batches. As shown in Figure 7A, it shows promising fabrication reproducibility with an RSD of 6.32%.

In addition, the storage stability of the SERS substrates is a crucial factor for practical applications. Herein, the long-term stability by SERS investigation is demonstrated in Figure 7B. Variation of SERS intensity recorded for 10 days is just 2.03%, showing excellent storage stability of this SERS substrate. Furthermore, the Fe_3_O_4_@Au also kept a commendable stability for ten days, as shown in Figure 7B. In all, magnetically inducing sandwich structure exhibits improved SERS sensing performance, including high sensitivity, signal homogeneity, acceptable preparation reproducibility, and long shelf-time stability.

### 3.4. Sensing Optimization

**Figure 8:**
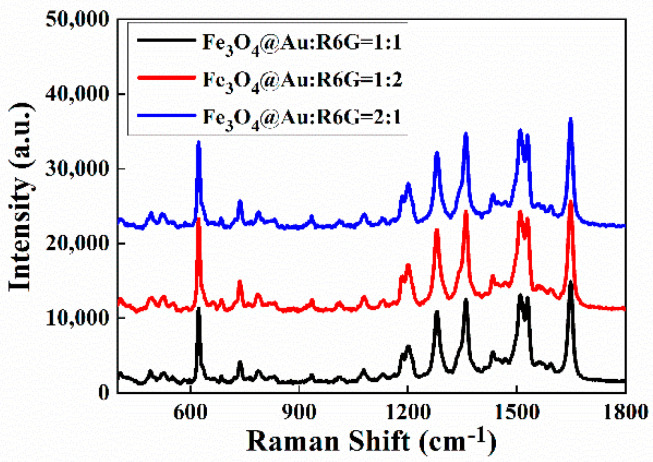
SERS spectra of 10^−6^ mol/L R6G solutions mixed with Fe_3_O_4_@Au NPs with different volume ratios, recorded on sandwich structures.

The different mixtures (volume ratios of 1:1, 1:2, and 2:1) of Fe_3_O_4_@Au and R6G (10^−6^ mol/L) were explored for reaching an optimal SERS sensing condition. As shown in Figure 8, the strongest Raman signal was obtained for the 1:2 volume ratio.

**Figure 9:**
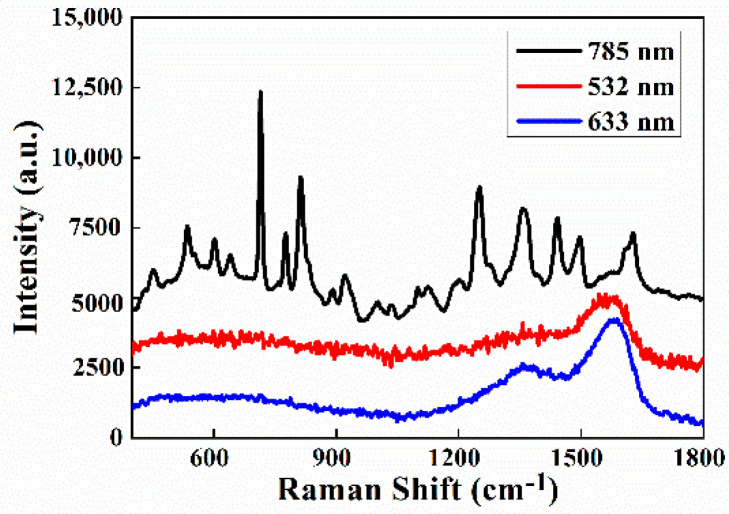
Sandwich-structure-based SERS spectra of MDA(100 μg/mL) solution under different excitation lasers.

It is well known that SERS intensities also depend on the excitation laser wavelength. Herein, three lasers with different wavelengths, 532, 633, and 785 nm, were used to record the SERS signals of MDA (100 μg/mL). As shown in Figure 9, when using 532 and 633 nm lasers, the characteristic Raman peaks of MDA could barely be observed due to visible laser thermal carbonization of the surface species. By contrast, excitation laser at 785 nm is a suitable option for the SERS experiment.

### 3.5. SERS Detection of MDA

**Figure 10:**
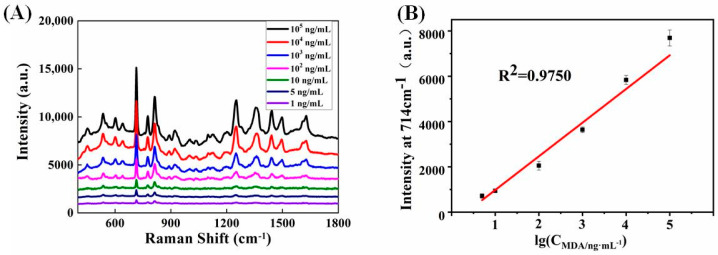
(**A**) Sandwich-structure-based SERS spectra of MDA with different concentrations. (**B**) Calibration plot based on Raman intensity at 714 cm^−1^.

By magnetically inducing sandwich-structure-based SERS sensing strategy, the quantitative detection performance of MDA was observed. As illustrated in Figure 10A, ultrasensitive detection of MDA with a minimum detection concentration of 1 ng/mL could be achieved. Figure 10B shows that a linear relationship between the denary logarithm of MDA concentrations in aqueous solutions and SERS intensities (*I*_714 cm_^−^^1^) could be obtained in the range from 5 to 10^5^ ng/mL with a reasonable correlation coefficient (*R*^2^ = 0.9750, and corresponding regression equation: y = 1486.253x − 507.167). The LOD value was estimated to be 0.0685 ng/mL according to the IUPAC standard method (Formula (1)):

LOD = 3 × RSD × BEC(1)

where RSD is the relative standard deviation of three replicates of the same experiment and BEC is the absolute value of the intercept between the linear regression equation and the *x*-axis. More recently, effective 1 October 2017, the Substance Abuse and Mental Health Services Administration (SAMHSA) established new testing criteria for MDA, for which the confirmation cutoff concentration is 500 ng/mL. Clearly, the LOD of the sandwich-structure-based SERS method is far below the required threshold. Therefore, the magnetically inducing sandwich-structure-based SERS sensing protocol is expected to be used during the initial period for inspecting or monitoring drug dependency.

**Figure 11:**
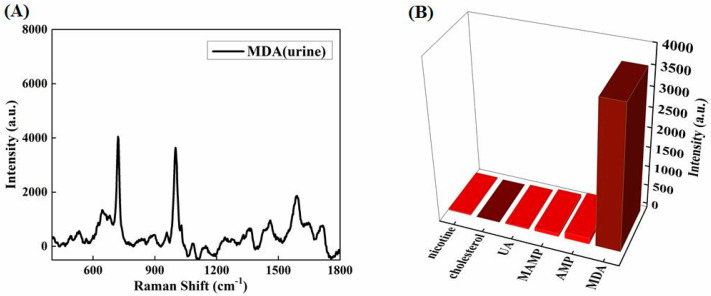
(**A**) Sandwich-structure-based SERS spectrum of MDA (1 μg/mL) in urine. (**B**) Intensities of the SERS band at 714 cm^−1^ for MDA and other interference species in urine samples.

For actual application, human urine from a health volunteer was spiked with a required amount of MDA standard solution. The characteristic peaks of MDA (1 μg/mL) in human urine can obviously be detected by the sandwich-structure-based SERS protocol, as shown in Figure 11A. Checking interference from bioactive molecules coexisting in complex physiological urine is particularly crucial for detection of MDA. As demonstrated in Figure 11B, the corresponding characteristic Raman band of MDA at 714 cm^−1^ is free from the interference of nicotine, cholesterol, uric acid (UA), methamphetamine (MAMP), and amphetamine (AMP). Consequently, by applying the sandwich-structure-based SERS assay, MDA can be easily distinguished in urine, which is beneficial to clinical and forensic monitors.

To validate the reliability of the SERS detection method, LC-MS as a standard method was used to detect MDA in the same urine sample. In Table 1, the good detection recoveries depict the acceptable reliability of the SERS method.

Moreover, we compared the detection results with other methods reported in the literature. As shown in Table 2, the sandwich-structure-based SERS strategy has the highest sensitivity and a wide concentration dynamic linear range. In short, compared to other methods, our sandwich-structure-based SERS strategy shows superior sensitivity, which is crucial for detecting low drug concentrations in biosamples.

## 4. Conclusions

In summary, a magnetically inducing sandwich structure was proposed for development of an SERS sensing platform through optimal preparation of ITO/Au substrate and Fe_3_O_4_/Au magnetic sorbs. Integrating the stability and homogeneity of a two-dimensional substrate of ITO/Au, and magnetic enrichment of Fe_3_O_4_/Au with magnetically inducing SPR hotspots, the novel SERS strategy exhibited ultrasensitive detection of MDA and good Raman signal reproducibility. Based on SERS intensity at 714 cm^−1^, the SERS detection of MDA presented a good linear relationship from 5 to 10^5^ ng/mL with LOD at 0.0685 ng/mL. In the future, the sandwich-based SERS protocol provides the possibility for rapid, sensitive, and reliable on-site detection of MDA.

## Figures and Tables

**Table 1:** Recovery and detection of MDA in urine by the sandwich-structure-based SERS strategy and the LC-MS method.

Spiked MDA (μg/mL)	SERS (μg/mL)	Recovery (%)	LC-MS (μg/mL)	Recovery (%)
1	0.9804	98.04	1.0260	102.6

**Table 2:** Comparison of the sandwich-structure-based SERS strategy for MDA with other reported methods.

Method	Linear Range	LOD	Real Sample	Reference
HPLC/FD	50–2000 ng/mL	10 ng/mL	Urine	[15]
GC-MS/MS	1–500 ng/mL	0.81 ng/mL	Urine	[16]
LC-MS	1–500 ng/mL	1 ng/mL	Oral Fluid	[18]
ELISA	–	8.2 ng/mL	Urine	[19]
Electrochemistry	0.61–400 ng/mL	0.36 ng/mL	Saliva	[46]
SERS	5–10^5^ ng/mL	0.0685 ng/mL	Urine	This work

## Data Availability

All data are contained within the article.
